# Human Tumor Cells Induce Angiogenesis through Positive Feedback between CD147 and Insulin-Like Growth Factor-I

**DOI:** 10.1371/journal.pone.0040965

**Published:** 2012-07-23

**Authors:** Yanke Chen, Xingchun Gou, Xia Ke, Hongyong Cui, Zhinan Chen

**Affiliations:** 1 College of Medicine, Xi’an Jiaotong University, Xi’an, China; 2 Department of Cell Biology & Cell Engineering Research Center & State Key Laboratory of Cancer Biology, Fourth Military Medical University, Xi’an, China; 3 Laboratory of Cell Biology & Translational Medicine, Xi’an Medical University, Xi’an, China; University of Hong Kong, Hong Kong

## Abstract

Tumor angiogenesis is a complex process based upon a sequence of interactions between tumor cells and endothelial cells. Previous studies have shown that CD147 was correlated with tumor angiogenesis through increasing tumor cell secretion of vascular endothelial growth factor (VEGF) and matrix metalloproteinases (MMPs). In this study, we made a three-dimensional (3D) tumor angiogenesis model using a co-culture system of human hepatocellular carcinoma cells SMMC-7721 and humanumbilical vein endothelial cells (HUVECs) in vitro. We found that CD147-expressing cancer cells could promote HUVECs to form net-like structures resembling the neo-vasculature, whereas the ability of proliferation, migration and tube formation of HUVECs was significantly decreased in tumor conditioned medium (TCM) of SMMC-7721 cells transfected with specific CD147-siRNA. Furthermore, by assaying the change of pro-angiogenic factors in TCM, we found that the inhibition of CD147 expression led to significant decrease of VEGF and insulin-like growth factor-I (IGF-I) secretion. Interestingly, we also found that IGF-I up-regulated the expression of CD147 in both tumor cells and HUVECs. These findings suggest that there is a positive feedback between CD147 and IGF-I at the tumor-endothelial interface and CD147 initiates the formation of an angiogenesis niche.

## Introduction

Most solid tumor growth relies on angiogenesis, which is a key event in tumor progression and cancer metastatic process [1]. The new vessels not only help to meet the growing metabolic demands of the tumor by supplying additional nutrients, but also provide potential routes for tumor dissemination and metastasis [2]. Angiogenesis is the result of the combined activity of the tumor microenvironment and signaling molecules. The angiogenic switch is represented as an imbalance between pro- and anti-angiogenic factors and is a rate-limiting step in the development of tumors [3]. During the initial phase, the following sequences of events usually occur [4]: genetic changes and local hypoxia in tumors contribute to increased secretion of soluble angiogenic factors by tumor cells, stromal cells and inflammatory cells. These angiogenic factors include: vascular endothelial growth factor (VEGF), basic fibroblast growth factors (bFGF), platelet derived growth factor (PDGF), epidermal growth factor (EGF), insulin-like growth factor (IGF), placental growth factor (PLGF), and so on. All these factors promote the sprouting of new vessels from nearby existing vessels. The autocrine and paracrine production of growth factors promoting angiogenesis ultimately function on endothelial cells (ECs). Normally, quiescent ECs are in contact with pericytes and a laminin-rich basement membrane. During angiogenesis, activated ECs loosen inter-cell contacts and secrete proteases to break down the surrounding basement membrane and extracellular matrix. ECs re-enter into the cell cycle, proliferate, migrate, and invade the surrounding stromal matrix. These ECs subsequently form a lumen and finally assemble to a new blood vessel [5].

The multistep process of angiogenesis involves serial interactions between tumor cells and ECs. Stimulation of ECs by tumor cells establishes an endothelial phenotype consistent with the initial stages of angiogenesis. This phenotypic switch can be modeled by co-cultivation of tumor and ECs. Routine cell culture systems without extracellular matrix (ECM) fail to provide a well-defined extracellular microenvironment for malignant tumor research [6], [7]. Thus, a 3D co-culture model of tumor cells with stromal cells in ECM was established to study the interactions between them and the influence of the microenvironment on cellular functions [7].

CD147 is a membrane glycoprotein expressed at varying levels in many cell types and is greatly enriched on the surface of tumor cells. CD147 levels are correlated with tumor progression and poor prognosis [8], [9], [10], [11]. Several studies have suggested that CD147 regulation of signal transduction initiated secretion of angiogenic factors by tumor cells and stromal cells in the tumor microenvironment. As a protease-inducer, CD147 could stimulate the surrounding fibroblasts and ECs to produce matrix metalloproteinases (MMPs) in autocrine and paracrine fashions [12], [13], [14], [15]. Recently, multiple studies have provided evidences that CD147 regulated tumor angiogenesis by stimulating MMPs and VEGF production in tumor and stromal cells [11], [16], [17], [18]. VEGF is expressed through alternative splicing as six different isoforms (VEGF_121_, VEGF_145_, VEGF_165_, VEGF_183_, VEGF_189_ and VEGF_206_). VEGF_121_, VEGF_165_ and VEGF_189_ are the predominant isoforms secreted by a wide range of normal and transformed cells [19]. Bougatef et al. have found that CD147 selectively increased the expression of soluble VEGF_121_ and VEGF_165_ isoforms, but not the VEGF_189_ form [18].

Although the role of CD147 in regulating cancer progression has been studied extensively, but little is known about the regulation of CD147 expression. Previous studies have reported that CD147 expression was upregulated under ischemic conditions in neuronal and cardiac cells [20], [21]. Recent studies have focused on the mechanisms underlying the CD147 overexpression, and reported the contributing roles of amphiregulin [22], cyclophilin 60 [23], cyclophilin A [24], transforming growth factor β [25] and **hypoxia** [26]. Our previous research has shown that the expression of CD147 was upregulated in active HUVECs after addition of exogenous growth factors, which further promoted angiogenesis by a direct effect on ECs [27]. But it is still unknown about which growth factor induce the expression of CD147 in active HUVECs. Here we investigated the role of CD147 in the multistep process of angiogenesis and examined the expression levels of CD147 in HUVECs after stimulation with VEGF, bFGF, EGF and IGF-I, respectively. We discovered a novel positive feedback regulatory mechanism controlling the interaction between tumor and HUVECs. Our results demonstrated that CD147 could positively regulate the secretion of IGF-I from tumor cells. What’s more, induced IGF-I positively counter- regulated CD147 expression in tumor and HUVECs.

## Materials and Methods

### Cell Cultures

Human hepatoma cell SMMC-7721, HepG-2, lung adenocarcinoma cell A549, and mammary carcinoma cell MCF-7 were provided by the Institute of Cell and Biochemistry, Chinese Academy of Sciences (Shanghai, China) and growed in RPMI 1640 medium supplemented with 10% fetal bovine serum (FBS) at 37°C under a mixture of 95% air and 5% CO_2_.

The original primary human umbilical vein endothelial cells (HUVECs) were isolated from human umbilical veins by using collagenase (Roche Diagnostics, Switzerland) as described by Bordenave et al [28] and maintained on 0.2% gelatin coated in EGM-2 medium (Lonza, Walkersville, MD, USA) which consists of endothelial cell basal medium (EBM-2) with 10% FBS and other additives (including VEGF, bFGF, EGF and IGF-I). HUVECs were used for experiments between Passages 2 and 5.

To induce hypoxia, cells were incubated at 5% CO_2_, and 1% O_2_ balanced with N_2_ using a sealed chamber at 37°C.

### RNA Interference

A specific siRNA targeting CD147 (5′-GUUCUUCGUGAGUUCCUCdTdT-3′, 3′-dTdTCAAGAAGCACUCAAGGAG-5′) (CD147 siRNA) and a negative control siRNA (control siRNA) were synthesized by Ambion Research Inc. (Austin, TX, USA). SMMC-7721 cells and HUVECs were transfected with 50 pM siRNA using the Lipofectamine 2000 reagent (Invitrogen, California, USA) according to the manufacturer’s protocol. Transfected cells were cultured for 24–48 h and then used for further experiment.

### *In vitro* Matrigel Three-dimensional Angiogenesis Assays

HUVECs and SMMC-7721 cells were suspended in EBM-2 and RPMI 1640 medium at a density of 1×10^5^ cells/mL respectively. Matrigel matrix (Becton Dickinson, New Jersey) was melted on ice for 24 h. For 3D co-culture experiments, equal volume (100 µl) of suspended SMMC-7721 cells or not, HUVECs and melted Matrigel matrix were mixed and seeded in 24-well plates. The 3D co-culture was incubated at 37°C for 40 minutes to form 3D spherules before being covered with RPMI 1640 medium (containing 10% FBS). The culture media was changed every two days. At different time points, cell morphogenesis was observed using an inverted phase-contrast microscope (Olympus CX41, Tokyo, Japan). Tube formation was quantified by counting branches from four representative fields per replicate. These experiments were performed in triplicate.

### Confocal Microscopy

Confocal microscopy techniques were performed as previously described [29]. The 3D co-culture in the plate was carefully washed once with PBSCM (PBS containing 1 mmol/L MgCl_2_ and 1 mmol/L CaCl_2_) and then was fixed in 2.7% paraformaldehyde for 20 min at room temperature. After washed twice with PBSCM, the 3D co-culture was equilibrated with 25% sucrose and embedded in Tissue-Tek OCT (McCormick, Norcross, USA). Then sections thickened in 15 µm were cut with a cryotome (LEICA CM1900, Wetzlar, Germany).

Immunofluorescence staining was performed to observe the morphous of both SMMC-7721 cells and HUVECs. After washed with PBS three times, the sections were blocked with 5% free fat milk for 30 minutes and then incubated with both the anti-human KDR/Flk rabbit monoclonal antibody (Chemicon, CA) and the anti-human CD147 mouse monoclonal antibody (prepared in our laboratory) for 45 minutes at 37°C. After washed with PBS three times, the sections were incubated with both Alexa-594 conjugated goat anti-rabbit secondary antibody (Pierce Biotechnology, Rockford, IL, USA) and FITC conjugated goat anti-mouse secondary antibody (Pierce Biotechnology, Rockford, IL, USA) for 30 minutes, subsequently were counterstained with DAPI (Biotium, Hayward, USA) for 1 min in the dark at room temperature. Finally, the sections were observed by using a laser scanning confocal microscope (Olympus FV1000, Tokyo, Japan).

### Collection of TCMs from SMMC-7721 Cells

After transfected with CD147 siRNA or control siRNA for 24 h, SMMC-7721 cells were split into two groups containing normoxia group which was cultured in 21% O_2_ and hypoxia group in 1% O_2_ for 12 h, respectively. Both of them were maintained in RPMI 1640 medium. Then TCMs were obtained from culture supernatant by centrifuged (250 g for 5 minutes) to remove floating cells.

### Removal of IGF-I from the TCMs

In order to remove IGF-I from the TCMs, the medium was incubated with anti-human IGF-I mouse monoclonal antibody (1 µg/mL) at 4°C for 12h and further incubated with Protein A-Sepharose beads link-coupled rabbit anti-mouse IgG at 4°C overnight. On the next day, Protein A-Sepharose beads were removed by centrifugation and the supernatants were collected. The removal effect of IGF-I in the TCMs was detected using ELISA assay kits (R&D Systems, Minneapolis, MN) according to the manufacturer’s protocol.

### *In vitro* 2D-Matrigel Endothelial Capillary-like Structure Formation Assay

After cultured in EBM-2 without FBS overnight, HUVECs were planted in 96-well plates (1×10^4^ cells/well) which was previously coated with 50 µl Matrigel (Becton Dickinson, BD). After 4 hours, capillary tube formation was observed with a phase contrast microscope (Olympus CKX41, Japan).

### TCMs Inducing-HUVEC Proliferation by BrdU Assay

The 5×10^3^ HUVECs were planted in gelatin-coated 96-well plates and cultured in EBM-2, EGM-2, or these above-mentioned TCMs respectively. The cell proliferation was measured using BrdU ELISA kit (Roche Applied Sciences) according to manufacturer’s instruction.

### *In vitro* Invasion Assay

The assay was done by using chambers with polycarbonate filters (pore size, 8 µm) coated on the upper side with Matrigel. HUVECs were detached and washed in PBS, suspended in serum-free EBM-2 medium containing 1% bovine serum albumin (BSA), and then added to the upper compartment of the chamber at a total number of 5×10^3^ cells per chamber. The lower compartment of the chambers was filled with various TCMs. After incubation for 12 or 24 hours, the number of invasing cells through the filter was counted after hematoxylin and eosin staining and plotted as the mean number of invasing cells per optic field in three independent experiments.

### VEGF, bFGF, EGF and IGF-I Assay by ELISA

The concentration of VEGF, bFGF, EGF or IGF-I in the TCMs was determined by using ELISA assay kits (R&D Systems, Minneapolis, MN) according to the manufacturer’s protocol. ELISA data was acquired by the use of a VersaMax Tunable MicroPlate Reader (Molecular Devices, Sunnyvale, CA) at 490 nm.

### Western Blot Analyses

Cells were lysed and protein concentrations were measured by the BCA Assay (Pierce Biotechnology). Total protein (10 µg) was separated on a 12% SDS-PAGE and transferred to polyvinylidene difluoride membranes (PVDF). The PVDF membranes were subsequently immunoblotted with the appropriate primary antibodies including anti anti-human CD147 mouse monoclonal antibody (prepared in our laboratory), anti-human actin mouse monoclonal antibody (Chemicon International, Inc) and anti-human HIF-1α mouse monoclonal antibody (Chemicon International, Inc). After extensive washed, the membranes were incubated with a horseradish peroxidase-conjugated goat anti-mouse secondary antibody (Sino-American Biotechnology Co., Luoyang, China). Signals were detected by an ECL kit (Pierce Biotechnology) according to the manufacturer’s instructions.

### Reverse transcription-polymerase chain reaction (RT-PCR) and Real-time Quantitative PCR (q-PCR)

Total RNA was isolated by the TRIzol reagent (Invitrogen) and reversely transcripted into cDNA with Superscript first strand synthesis kit (Invitrogen) according to the manufacturer’s protocol. VEGF isoforms (121, 165, 189 and 206) and glyceralde hyde-3-phosphate dehydrogenase (GAPDH) mRNA expression levels were assayed by using RT-PCR. CD147, HIF-1α, IGF-I, β-actin mRNA expression levels were measured by qPCR using MiniOpticon real-time PCR detection system (Bio-Rad, Hercules, CA, USA) in SYBR Green master mix (Takara, Otsu, Japan) according to the manufacturer’s protocol. All data were analyzed by using Opticon Monitor software (version 3.1; Bio-Rad). All primers were synthesized by the Shanghai Sangon Biological Engineering Technology and Services Co. Ltd (Shanghai, China). The sequences of PCR primers are listed:

VEGF: 5′-CTCACCGCCTCGGCTTGTCACA-3′,

5′-CCTGGTGGACATCTTCCAGGAGTA-3′;

GAPDH: 5′-GGTGGTCTCCTCTGACTTCAACA-3′,

5′-GTTGCTGTAGCCAAATTCGTTGT-3′;

HIF-1α: 5′-TCGACACAGCCTGGATATGA-3′, 5′-CGGCTGCGGCCAGCAAAGTT-3′;

CD147: 5′- TCGCGCTGCTGGGCACC-3′,

5′- TGGCGCTGTCATTCAAGGA -3′;

IGF-I: 5′- GCAATGGGAAAAATCAGCAG-3′,

5′-GAGGAGGACATGGTGTGCA-3′;

β-actin: 5′- GGAAATCGTGCGTGACATT -3′,

5′- GACTCGTCATACTCCTGCTTG -3′.

### Statistical Analysis

The statistical significance of the results was determined by using the Student’s t-test. A value of *P*<0.05 was considered significant. The means ± SD values of three experiments are presented, and the differences between the experimental conditions were evaluated using the ANOVA analysis (*P*<0.05 was considered significantly).

## Results

### Morphological Change of HUVECs and SMMC-7721 Cells in 3D Co-culture Models

**Figure 1:**
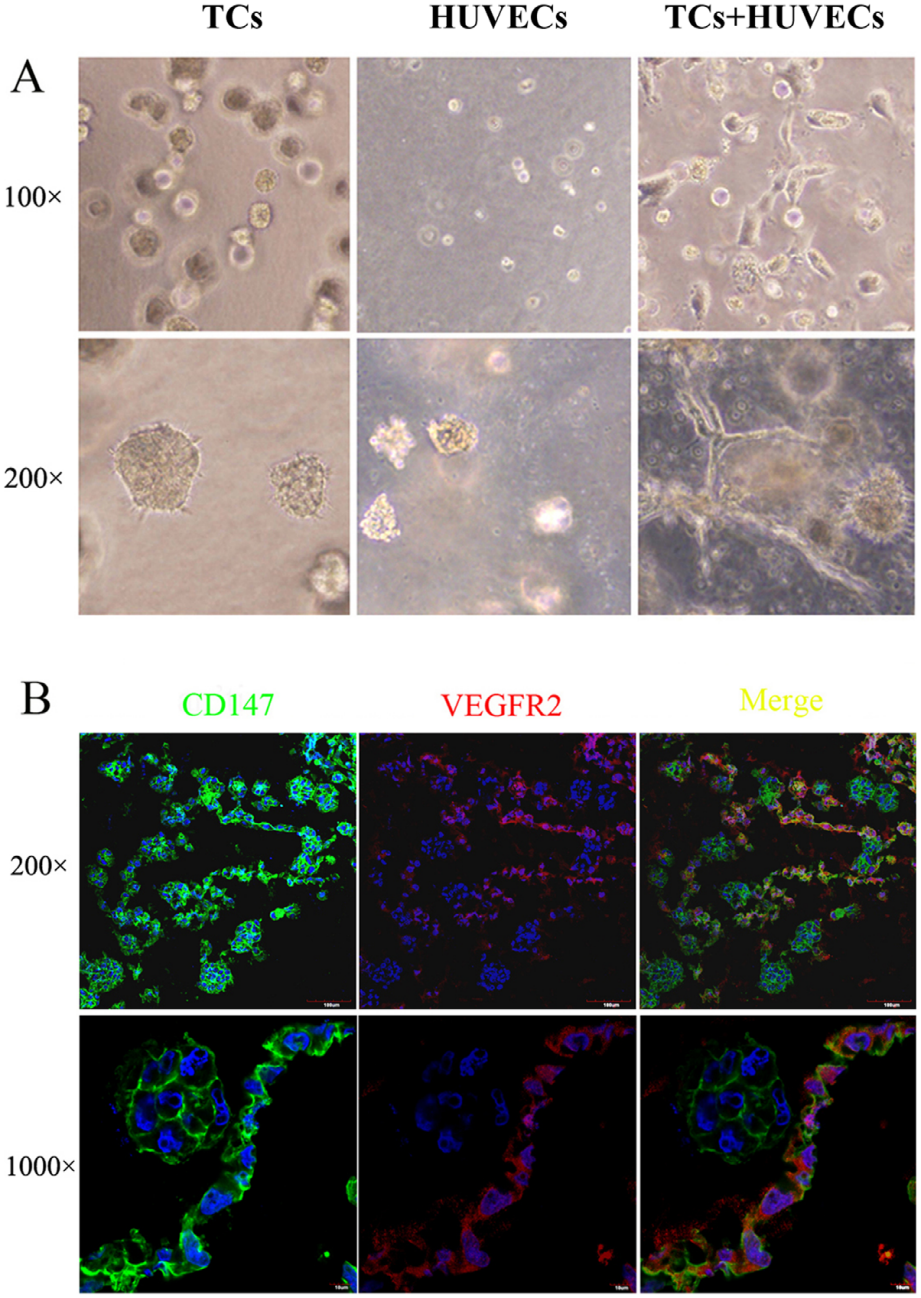
Morphological change of SMMC-7721 cells and HUVECs in a 3D co-culture model. (A) SMMC-7721 cells and HUVECs were seeded and cultured in Matrigel. Morphogenesis was observed by using an inverted phase-contrast microscope. (B) These 3D-cultured cell sheets were fixed and cut into sections which were stained with anti CD147 (green) and KDR/Flk (red) antibodies, and DAPI (blue), respectively, and observed using a laser scanning confocal microscope.

In the 3D angiogenesis models, HUVECs and/or SMMC-7721 cells were seeded in the Matrigel with the medium added to the top of the gel before polymerization. As shown in Fig. 1A, in the SMMC-7721 cells group, we can see that large, spheroidal and unpolarized invasive colonies clusters gradually formed after 24 h culture (Fig. 1A left graph). Whereas in the group of HUVECs which were cultured in EBM-2 medium without growth factors, the cells were still single and some began to die after 3 days culture (Fig. 1A middle graph ). However, when HUVECs and SMMC-7721 cells were incubated together, the HUVECs generally proliferated and elongated to form threadlike tubular networks without any additional growth factors (Fig. 1A right graph). These results suggested SMMC-7721 cells induced angiogenesis in 3D co-culture model.

The 3D co-culture sections were double-stained with anti-CD147and KDR/Flk antibodies, counterstained with DAPI and then observed by a laser scanning confocal microscope. CD147(green) expressed in both HUVECs and SMMC-7721, KDR/Flk (red) was observed in only HUVECs (Fig. 1B).

### Knock-down of CD147 in SMMC-7721 Cells Inhibited the Vascular Formation of HUVECs *in vitro*

**Figure 2:**
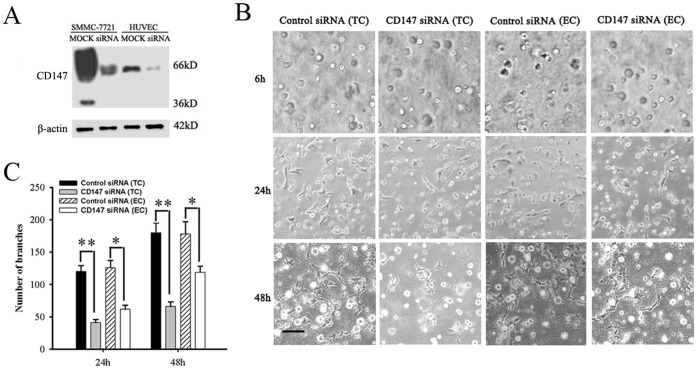
The angiogenic phenotype was inhibited by knock-down of CD147 in either SMMC-7721 cells (TC) or endothelial cells (EC) in a 3D co-culture model. (A) CD147 expression was analyzed by western blot in SMMC-7721 cells and HUVECs after transfected with CD147 siRNA or control siRNA for 48 h. (B) *In vitro* tube formation analysis in Matrigel. After SMMC-7721 cells and HUVECs were transfected with CD147 siRNA or control siRNA respectively, SMMC-7721 cells were co-cultured with HUVEC cells for 48 h in Matrigel. Images were acquired at 6, 24 and 48 h, using an inverted microscope (Olympus CKX41) fitted with a 10× phase-contrast objective lens. (C) Semi-quantitative assessment of tube formation was performed by determining the number of branches per field. Results are based on four randomly selected fields and are expressed as the mean ± SD of three independent experiments. Statistical significance was determined by the Student’s t-test. *P<0.05, **P<0.001, compared with the control level.

To investigate the role of CD147 during the process of angiogenesis, either SMMC-7721 cells or HUVECs were respectively transfected with CD147 siRNA or control siRNA and co-cultured in Matrigel. As shown in Fig. 2B (left graph), HUVECs began to sprout after 24 h culture and a net-like pattern was formed after 48 h culture in control siRNA group. However, the sprouting and formation of microvessel-like structures were markedly decreased when SMMC-7721 cells were transfected with CD147 siRNA (*P*<0.01) after both 24 h and 48 h culture (Fig. 2B secondary series and Fig. 2C). The microvessel-like structures also decreased when HUVECs were transfected with CD147 siRNA (*P*<0.05).These data suggest that CD147 could induce HUVECs migrating toward SMMC-7721 cells and enhance HUVEC vascular formation.

### Regulation of Tumor Cell Associated CD147 in the Proliferation, Migration and Capil2lary-like Structure Formation of HUVECs

In order to elucidate the role of CD147 on angiogenesis through paracrine, SMMC-7721 cells were transfected with CD147 siRNA. After they were cultured in normoxia or hypoxia for 12h, their culture supernatants (i.e. TCMs) were collected. Next, we analyzed the proliferation, migration and tube formation of HUVECs when they were cultured in TCMs.

**Figure 3:**
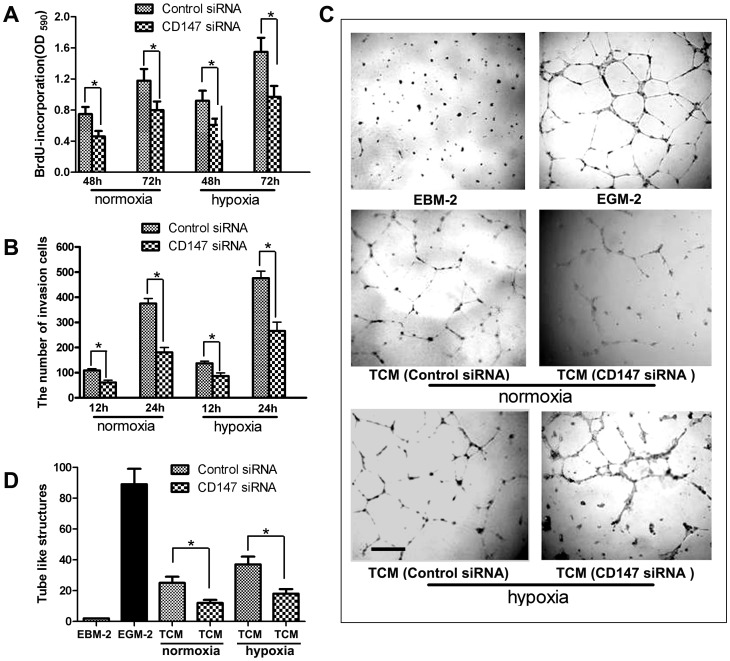
HUVEC proliferation, migration and formation of capillary-like structures were assessed. HUVECs were cultured with TCMs obtained from SMMC-7221 cells transfected with CD147 siRNA or control siRNA in either normoxia or hypoxia conditions for 24 h. (A) HUVECs proliferation was measured using the BrdU incorporation assay. HUVECs were cultured with these TCMs for 3days. The data were presented as absorbance at 490 nm (mean ± SD of three replicate experiments). *P<0.05, compared with the control level. (B) The migration ability of HUVECs was examined by the *in vitro* invasion assay. Cells were first planted on the upper compartment of the Boyden chambers; the lower compartment was filled with various TCMs. After 12h and 24 h of incubation, the number of cells migrating through the filter was counted and plotted as the mean number of migrating cells in an optic field in three independent experiments. *P<0.05, compared with the control level. (C) and (D) The formation of capillary-like structures were induced in different TCMs. HUVECs were seeded on top of the Matrigel and cultured for 4 h with TCMs which were mixed with EBM-2 in a 1∶1 ratio. The formation of capillary-like structures was photographed in (C), and Semi-quantitative assessment of tube formation was performed by counting the number of branches per field. *P<0.05, compared with the control level.

As shown in Fig. 3A, B, the results suggested that both proliferation and invasion of HUVECs were considerably inhibited when HUVECs were cultured in the TCM from culture supernatant of SMMC-7721 cells transfected with CD147 siRNA, compared with the TCM from SMMC-7721 cells which transfected with control siRNA regardless of normoxia or hypoxia condition. Furthermore, in the *in vitro* 2D Matrigel tube formation assay, HUVECs formed a significantly reduced number of branches when they were cultured in the TCM from SMMC-7721 cells transfected with CD147 siRNA (Fig. 3C and D). We also found that this induced effect of the TCM from hypoxia treated cells was higher than the TCM from normoxia treated cells (Fig. 3).Taken together, these results indicated that CD147 maybe an induced pro-angiogenesis factor which promotes HUVEC proliferation and migration.

### Decrease of VEGF and IGF-I by Knock-down of CD147 in SMMC-7721 Cells

**Figure 4:**
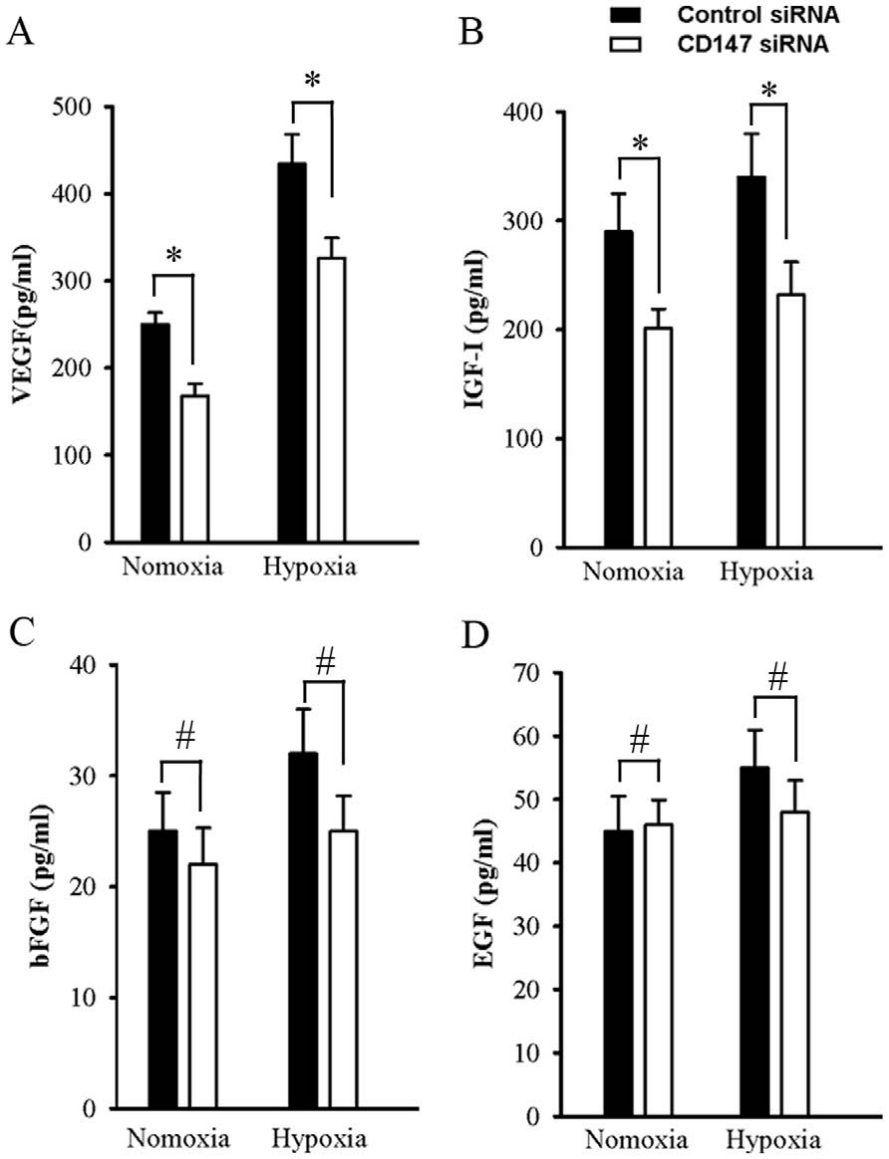
Knock-down of CD147 in SMMC-7721 cells decreased the levels of VEGF and IGF-I in TCMs under normoxia and hypoxia conditions. All figures are a representation of three trials. The concentrations of VEGF (A), IGF-I (B), bFGF (C) and EGF (D) in TCMs were determined by ELISA analysis. *P<0.05, ^#^ *P*>0.05, compared to the control level.

To determine whether CD147 could induce secretion of pro-angiogenic factors (e.g. VEGF, bFGF, EGF and IGF-I) in a paracrine manner or not, we measured the level of these factors in the TCMs by using ELISA. The concentration of VEGF and IGF-I were significantly lower in the TCMs from SMM7721 transfected with CD147 siRNA than control siRNA under either normoxia or hypoxia conditions (Fig. 4A, 4B). Under normoxia condition, knock-down of CD147 resulted in a decrease of VEGF secretion from 252.5±13.5pg/mL to 168.0±12.8 pg/mL, and IGF-I secretion from 290±35.1pg/mL to 201±23.2 pg/mL; under hypoxic condition, knock-down of CD147 resulted in a decrease in VEGF secretion from 434.6±34.0pg/mL to 326.1±23.7pg/mL, and IGF-I secretion from 340.5±35.1pg/mL to 242.1±25.3 pg/mL. But levels of bFGF and EGF were very low in the TCM under both normoxia and hypoxia conditions, also, knock-down of CD147 did not significantly change the concentration of these two growth factors in the TCM (Fig. 4C, 4D).

### Decrease of VEGF, IGF-I and HIF-1α by Knock-down of CD147 in SMMC-7721 Cells

**Figure 5:**
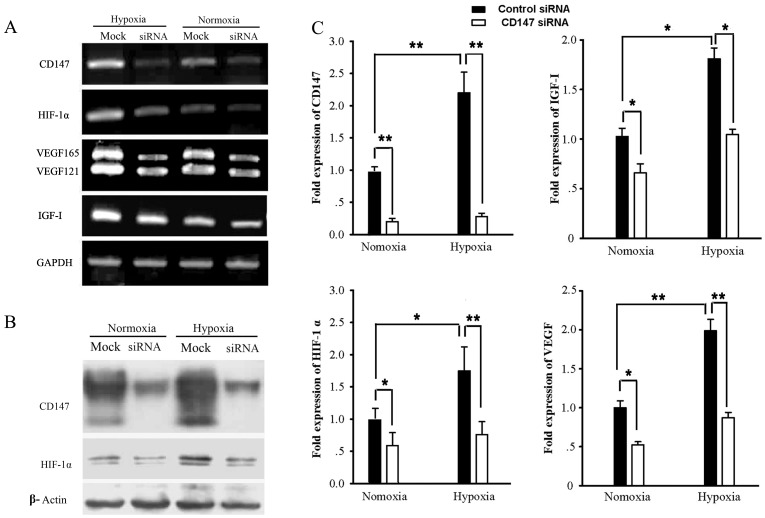
Knock-down of CD147 down-regulated the expression of VEGF, IGF-I and HIF-1α in normoxia and hypoxia conditions. All figures are a representation of three trials. (A) RT-PCR was performed to examine the transcription levels of CD147, VEGF, IGF-I, HIF-1α and GAPDH in SMMC-7721 cells transfected with CD147 siRNA or control siRNA. (B) Western blots were performed to examine the expression level of CD147, HIF-1α, and β-actin proteins in SMMC-7721 cells transfected with CD147 siRNA or control siRNA. The expression of β-actin was used as internal control. (C) qRT-PCR was performed to examine the transcription levels of these molecular in SMMC-7721 cells transfected with CD147 siRNA or control siRNA. *P<0.05, **P<0.001, compared with the control level.

HIF-1α is considered to be a key regulator of VEGF gene expression, so we wanted to determine whether knock-down of CD147 would change the expression of HIF-1α or not. The data in Fig. 5A and B showed that knock-down of CD147 down-regulated the expression of HIF-1α at both RNA and protein levels. RT-PCR was performed to detect RNA expression of VEGF isoforms (121, 165, 189 and 206) with specific common primer pair, but only VEGF_121_ and VEGF_165_ were observed and decreased significantly in CD147 knock-down SMMC-7721 cells under either normoxia or hypoxia conditions(Fig. 5A). Further, we found that IGF-I was also significantly lower in SMMC-7721 transfected with CD147 siRNA than control siRNA. The qRT-PCR also confirmed the above results (Fig. 5C).

### Up-regulation of CD147 by IGF-I in Both HUVECs and Tumor Cells

**Figure 6:**
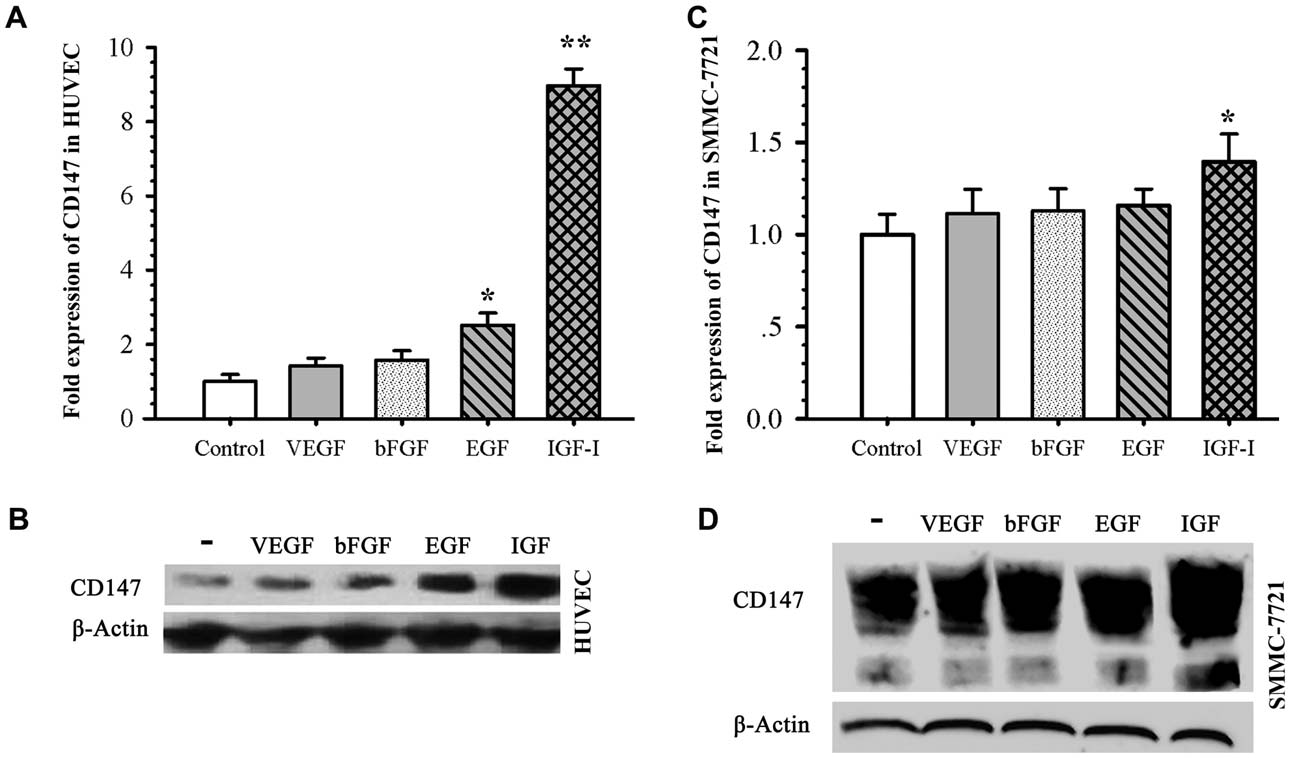
IGF-I induced CD147 expression in HUVECs and SMMC-7721 cells. CD147 expression level was determined by qRT-PCR and western blot in HUVECs (A and B) or SMMC-7721 (C and D) which were treated with VEGF, bFGF, EGF and IGF-I (100ng/mL) for 24 h. *P<0.05, **P<0.0001, compared with the control level.

To investigate which of these factors (VEGF, bFGF, EGF and IGF-I) up-regulates CD147 in activated HUVECs thereby promoting angiogenesis, CD147 expression was determined in HUVECs treated with VEGF, bFGF, EGF and IGF-I respectively for 24 h by qRT-PCR and western blot analysis. We observed both EGF and IGF-I strongly up-regulated the expression of CD147 in HUVECs, and the role of IGF-I was stronger than EGF. But VEGF and bFGF had a minor stimulatory effect on CD147 expression (Fig. 6A, B). In SMMC-7721 cells, only IGF-I remarkably up-regulated the expression of CD147 in RNA level (Fig. 6C) and protein level (Fig. 6D).

Furthermore, to investigate whether the CD147 expression induced by IGF-I is dose-dependent in tumor cells, the expression of CD147 was determined in the SMMC-7721, HepG-2, A549,and MCF-7 cells treated with different concentration of IGF-I respectively.

**Figure 7:**
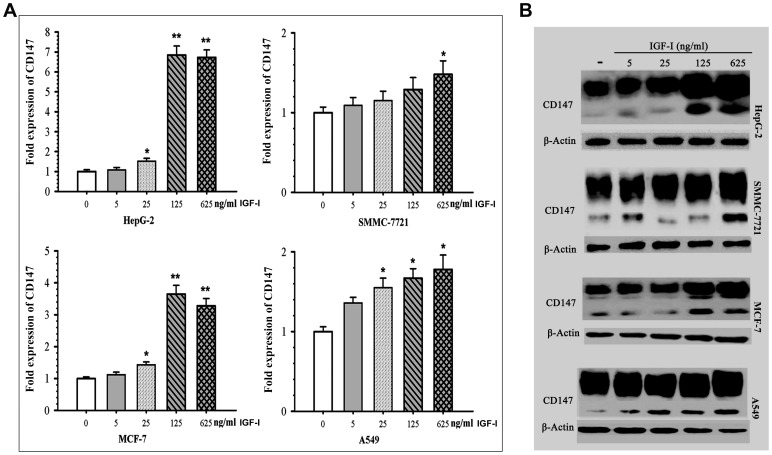
IGF-I induced CD147 expression in dose-dependent fashion in multiple tumor cells. (A, B) qRT-PCR (A) and Western blot (B) analysis of CD147 expression level in SMMC-7721, HepG-2, A549, and MCF-7 cells in response to stimulation by IGF-I for 24 h.

The qRT-PCR analysis showed that IGF-I up-regulated CD147 mRNA expression in a dose-dependent manner and the concentration of 625ng/mL exhibited the greatest stimulation effect in these tumor cells (Fig. 7A). Western blot showed IGF-I up-regulated CD147 expression in dose-dependent fashion in HepG2 and MCF-7 cells; in SMMC-7721 cells and A549 cells, only low glycosylation of CD147 was significantly up-regulated response to IGF-I stimulation (Fig. 7B).

Taken together, these results suggested that the IGF-I maybe an important regulatory element of CD147 expression in HUVECs and many kinds of tumor cells.

### IGF-I was a Specific Up-regulator of CD147 Expression to Promote Angiogenesis

**Figure 8:**
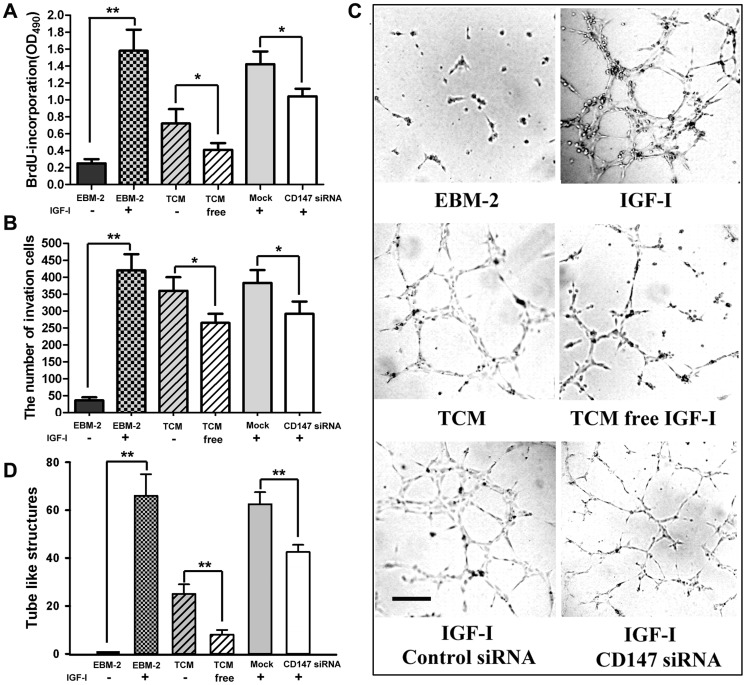
IGF-I was a specific up-regulator of CD147 expression to promote angiogenesis. We removed IGF-I from TCMs by using immunoprecipitation and observed the change of proliferation (A) and migration (B) and formation tube-like structures (C and D) of HUVECs response to TCM, IGF-I and siRNA CD147.

To observe whether IGF-I was a specific up-regulator of CD147 express to promote angiogenesis, we removed IGF-I from TCMs by immunoprecipitation. ELISA analysis revealed that IGF-1 was barely detectable in TCMs. We found that the ability of proliferation, migration and formation tube-like structures of HUVECs was significantly enhanced in response to IGF-I and TCMs compared with EBM-2, but removal of IGF-I from TCMs decreased the inductive effect for HUVECs (Fig. 8). In addition, the application of CD147 siRNA significantly inhibited proliferation, migration and angiogenesis of HUVECs in response to IGF-I.

## Discussion

The microenvironment is now being viewed as one of the critical elements which promotes the transition from carcinoma in situ to invasive cancer. Cells and extracellular matrix molecules are intimately involved in tumor angiogenesis [30]. Previous studies have shown that interactions between tumor cells and surrounding stromal cells are mediated by CD147. Millimaggi et al. reported that CD147 expresses in microvesicles derived from epithelial ovarian cancer cells and CD147-positive vesicles promote an angiogenic phenotype in ECs *in vitro* [31]. Another study reported by Tang et al. showed that tumor cell-associated CD147 stimulated its own expression in the tumor stroma by a positive feedback regulation model, consequently to contribute to tumor angiogenesis, tumor growth, and metastasis [32].

In the present study, our results showed that in a 3D co-culture system, solitary HUVECs cultured could not proliferate quickly or form threadlike tubular networks in EBM media without required growth factors. When both HUVECs and SMMC-7721 cells were co-cultured, SMMC-7721 cells activated HUVECs and promoted HUVECs’ proliferation and invasion. The knock-down of CD147 in either SMMC-7721 cells or HUVECs significantly inhibited angiogenesis.

To further explore the potential regulatory mechanism of CD147 in inducing angiogenesis, we collected the TCMs of SMMC-7721 cells which were transfected with CD147 siRNA or control siRNA respectively. We found that the stimulatory effects of the TCMs on HUVECs were significantly reduced when CD147 expression was inhibited in SMMC-7721 cells. Data from these experiments suggested that CD147 promoted angiogenesis through a paracrine manner.

Hypoxia is the major pathophysiological condition which regulates angiogenesis. HIF-1, a key hypoxia response transcription factor, induces the expression of several target genes, including VEGF [33], [34], [35], bFGF [36], and IGF [37]. VEGF regulates the functions of ECs as confirmed by knockout of VEGF *in vitro* and *in vivo* [38], [39]. CD147 promoting angiogenic through HIF-1α, HIF-2α and VEGF pathways has already been reported in various tumors [16], [17], [18]. Recent study showed that CD147 regulated VEGF expression via the PI3K-Akt and MAPK (mitogen-activated protein kinase)-dependent signaling pathway [16], [40]. In our study, we also confirmed that hypoxia could induce the expression of HIF-1, VEGF, IGF-I, and we reported in the first time that hypoxia also induce the expression of CD147 which in turn induced secretion of VEGF and IGF-I, which indicated that CD147 made more important role in angiogenesis under hypoxia condition.

At the same time, we also found that IGF-I strongly induced the expression of CD147 in HUVECs cells and multiple tumor cell lines, which showed that there was a positive feedback mechanism between CD147 and IGF-I. It is known to all that IGF-1 is an important mitogenic and antiapoptotic peptide, which affects the proliferation of normal and malignant cells and elevated levels of IGF-I are associated with carcinogenesis and the progression of cancer [41], [42]. In order to elucidate the role of IGF-I on angiogenesis, we removed IGF-I from TCMs and subsequently observed the tube-like structures formation. The result showed that IGF-I strongly induced angiogenesis, which was partly blocked by CD147 siRNA. So CD147 is a key regulator of angiogenesis, and this effect of CD147 is likely mediated by IGF-I.

In conclusion, the tumor-microenvironment may play a crucial role in facilitating tumor angiogenesis. Tumor-associated CD147 actively mediates the cross-talk between tumor and endothelial cells, and may promote angiogenesis by regulating the tumor-stromal microenvironment which contains pro-angiogenic factors such as MMPs, VEGF, IGF-I and CD147 itself. As a consequence, tumor cells recruit ECs and stimulate their proliferation, migration, and tube formation. In particular, IGF-I up-regulated the expression of CD147 in tumor cells and ECs which further promoted tumor angiogenesis. These results have a direct impact on cancer therapy by inhibiting tumor angiogenesis through the inhibition of CD147.
